# FERM domain containing protein 7 (FRMD7) upregulates the expression of neuronal cytoskeletal proteins and promotes neurite outgrowth in Neuro-2a cells

**Published:** 2012-06-01

**Authors:** Jiali Pu, Xiaoxiong Lu, Guohua Zhao, Yaping Yan, Jun Tian, Baorong Zhang

**Affiliations:** Department of Neurology, second Affiliated Hospital, College of Medicine, Zhejiang University, Hangzhou, Zhejiang, China

## Abstract

**Purpose:**

Mutations of the FERM domain containing protein 7 gene (*FRMD7*) are associated with X-linked idiopathic congenital nystagmus. Previous studies have shown that FRMD7 plays an important role in neuronal development and is involved in the regulation of F-actin. However, its specific mechanism of action remains undetermined.

**Methods:**

Our study used quantitative real-time PCR to assess the levels of neuron-specific genes in a mouse neuroblastoma cell line (Neuro-2a) after transfection with a full-length coding transcript of *FRMD7* or a blank control vector. F-actin was detected by rhodamine-phalloidin staining. Neurite number and length were assessed by a confocal laser scanning microscope.

**Results:**

We demonstrated that *FRMD7* can promote neurite outgrowth following retinoic acid–induced differentiation in Neuro-2a cells. Neurites were significantly longer in cells transfected with *FRMD7*, but there was no difference in cell numbers. The mRNA expression of neuron cytoskeletal-related genes (microtubule-associated protein 2 [*Mtap2*], neurofilament-L and M [*NF-L* and *NF-M*] and the microtubule-associated protein tau [*MAPT*]) were significantly increased compared to controls. Other genes (*NF-H*, MAPT, neuron-specific class III beta-tubulin (*Tuj-1*), nestin, and growth-associated protein-43 [*GAP-43*]) were not obviously altered by *FRMD7* overexpression.

**Conclusions:**

Taken together, our data suggest that FRMD7 promotes the extension of neurites and may be involved in regulating the movement of cytoskeletal proteins, which influences not only F-actin, but also NF and microtubule dynamics.

## Introduction

Idiopathic congenital nystagmus (ICN) is an oculomotor disorder that arises from a primary defect in the brain regions involved in ocular motor control. ICN is characterized by involuntary horizontal oscillations of both eyes, and its onset occurs within six months of birth [1]. Patients with ICN experience a significant decrease in their quality of life over their lifetime. The prevalence of the disorder is estimated to be 24 per 10,000; pharmacological agents such as memantine and gabapentin have been shown to be effective for congenital nystagmus [2,3].

Mutations of the FERM domain containing protein 7 (*FRMD7*) gene are known to be one of the main causes of X-linked idiopathic congenital nystagmus. To date, more than 40 mutations of *FRMD7* have been reported from various ethnic backgrounds [4-9]. Previous studies have shown that downregulation of the expression level of *FRMD7* leads to a significant reduction in the length of neurites during retinoic acid (RA)-induced differentiation in Neuro-2a cells. Meanwhile, a notable disturbance in the dynamics of F-actin and F-actin/G-actin has been observed when *FRMD7* was knocked down in Neuro-2a cells [10]. Previous studies have shown that COOH-terminal truncated mutations alter their subcellular localization and lead to no longer co-localization with F-actin like the wild type FRMD7 [11]. This evidence suggests a critical role of FRMD7 in neurite outgrowth, which may be at least in part associated with the dynamics of F-actin. However, whether FRMD7 can directly trigger the initiation of neurite extension has not been elucidated. The precise biochemical role of FRMD7 in neuronal development, especially its involvement in ICN pathogenesis, remains unclear.

In vivo, soon after migrating to their final destination, neurons begin to form neurites. These neurites eventually differentiate to develop axons and dendrites [12]. Neuro-2a neuroblastoma cells have been widely used as an in vitro neuronal differentiation model that closely mimics the events that occur during neuronal development by incubation with RA [13,14]. RA-induced Neuro-2a cell differentiation leads to the expression of neurite-bearing and neuron-specific proteins, such as microtubule-associated protein 2 (Mtap2) and neuron-specific nuclear protein (NeuN), among others, which results in the development of neuron-like cells. An understanding of these neuron-specific genes, especially cytoskeletal-related genes, and the modification of their expression induced by the consequent overexpression of *FRMD7* in Neuro-2a cells, may reveal a novel link between *FRMD7* and neuronal outgrowth. In this study, we investigated neurite outgrowth and the expression levels of some neuron-specific proteins following overexpression of the protein FRMD7 in Neuro-2a cells.

## Methods

### Animals

The mice we used were purchased from the Animal Center of Zhejiang University School of Medicine (Hangzhou, China). All experimental procedures were approved by the institutional committee at Zhejiang University.

### Ribonucleic acid isolation and reverse transcription–polymerase chain reaction

Total RNAs were isolated using TRIZOL reagent (Invitrogen, San Diego, CA) according to the manufacturer’s instructions from 18-day embryonic mouse brains and Neuro-2a cells had previously been transfected with mFRMD7-pcDNA3.1(+) or pcDNA3.1(+) plasmids which were performed as follows (in the plasmid construction section). RNA (5 µg) was reverse transcribed using oligo dT by reverse transcriptase. Briefly, cells and tissues grounded to powder in liquid nitrogen were added the TRIZOL reagent to dissociation of nucleoprotein complexes. The Chloroform was used to separate the RNA exclusively in the aqueous phase. RNA was precipitated by Ethanol. RNA (5 μg) was reverse transcribed using oligo dT by reverse transcriptase. For PCR amplification, specific oligonucleotide primer pairs (10 pmol each) were incubated with 2 µl cDNA template in 25 µl PCR reaction mixtures containing 1.5 mM MgSO_4_, mixed deoxynucleotides (1 mM each), and 0.5 U KOD FX PLUS (Toyobo, Osaka, Japan) polymerase. The sequences of primers used were as follows: mouse *FRMD7* sense primer, 5'-ATG CTC CAT TTA AAA GTG-3' and antisense primer, 5'-AGG CTA AGA AAT AAT TGC-3' (product length, 2,112 bp). Dilutions of the cDNAs were amplified for 38 cycles at 94 °C for 2 min, 98 °C for 15 s, 60 °C for 45 s, and 68 °C for 2 min 20 s. The amplified PCR products were analyzed by 1.2% agarose gel electrophoresis and ethidium bromide staining.

### Plasmid construction

The PCR products were confirmed by subcloning the amplified cDNAs into the pGEM-T Easy Vector System (Promega, Madison, WI) for sequencing. Mouse *FRMD7* cDNA, was digested with XhoI and BamHI, and fused to enhanced green fluorescent protein (EGFP) in pEGFP-N1 (Invitrogen, Carlsbad, CA). The cDNA was digested with BamHI and XhoI, and subcloned into pcDNA3.1(+) vector (Invitrogen).

### Cell culture, transient transfection, and differentiation

Mouse neuroblastoma cells (Neuro-2a) were purchased from the Chinese Academy of Sciences Committee Type Culture Collection Cell Bank/CAS Shanghai Institutes for Biologic Sciences Cell Resource Center (Shanghai, China). Neuro-2a cells were cultured in Dulbecco’s modified Eagle’s medium (DMEM; Invitrogen) containing 10% fetal bovine serum (FBS; Invitrogen), and 10% penicillin and streptomycin. Cultures were maintained in 5% CO_2_ at 37 °C, and were passaged every 2–3 days. For differentiation experiments, cells were grown on chamber slides to 40%–50% conﬂuence in 24 well plates, then transfected with 0.4 µg plasmid DNA (mFRMD7-EGFP-n1 or pEGFP-n1) and 1.5 µl Attractene Transfection Reagent (Qiagen, Valencia, CA) per well. After 6 h, cells were rinsed and fresh DMEM (containing 10% FBS) was added. Twenty-four hours later, cells were subjected to RA (Sigma-Aldrich, St Louis, MO) treatment (10 µM RA in 2% FBS/DMEM).

### Western blotting, immunofluorescence, and F-actin staining

For western blots, cultured cells were homogenized in radioimmunoprecipitation assay buffer with 1× protease inhibitor cocktail (Sigma-Aldrich) and 100 mg/ml of phenylmethylsulfonyl fluoride. Equal amounts of protein were separated by sodium dodecyl sulfate PAGE (8% gels) and transferred to a polyvinylidene fluoride membrane (Bio-Rad, Hercules, CA). After blocking with 5% skim milk powder, membranes were incubated with the primary mouse anti-flag antibody (Sigma–Aldrich) at 1:4,000 dilution, and the membrane-bound antibody was visualized with horseradish peroxidase–conjugated secondary antibody (Abmart, Shanghai, China), diluted 1:5,000. The membranes were processed using the ECL advance western blotting detection kit (Qiagen). β-actin (Santa Cruz, CA) was used as a loading control.

Three days after RA treatment, cells were fixed in 4% formaldehyde in PBS (50 mM NaPO_4_, 150 mM NaCl, pH 7.4) for 15 min at room temperature (RT). F-actin was stained using TRITC-conjugated rhodamine-phalloidin (77481; Sigma-Aldrich) fluorescein diluted with PBS and BSA for 45 min at RT. After washing in PBS, cell nuclei were stained with 4',6-diamidino-2-phenylindole (1:5,000 dilution; ZhongShan Goldenbridge Biotechnology Co. Ltd., Beijing, China) for 5 min at RT. Morphological features were quantified using a confocal laser scanning microscope (Leica TCS SP5 X; Leica, Wetzlar, Germany).

### Neurite length measurement

Neurite length measurement was performed as described previously [15]. Neurite length was observed 72 h after RA-induced differentiation of Neuro-2a cells. Images were captured on a Leica TCS SP5 X microscope. Statistics were gathered on the percentage of cells with neurites (a neurite is defined as an outgrowth with a length more than half the diameter of the cell body) and longest neurite length (neurite length from cell body to distal tip), counted from at least five randomly taken images per well. The data presented are the mean of three independent experiments, shown as the mean±standard error of the mean (SEM), with p<0.05 via Mann–Whitney U test. At least 250 cells per experiment were scored for neurite outgrowth (Table 1).

**Table 1:** Neurite outgrowth of Neuro-2a cells treated with 10 uM retinoic acid.

**Measure items**	**control**	**FRMD7**
Cells with neurites (%)	38.63±3.65	41.87±3.46
Neurite length (μm)	68.99±2.59	94.40± 2.62**

Neuro-2a cells transfected FRMD7 or not were treated with 10 μm retinoic acid, 2% FCS/DMEM for 3 days, and neurites parameters were quantified. Datas are mean±SEM of three independent experiments. n>250 cells counted for each experiment. **p<0.01 compared with the control.

### Quantitative real-time polymerase chain reaction

Quantitative real-time (RT)-PCR amplification was performed to assess the relative abundance of neuron-specific genes (microtubule-associated protein 2 (*Mtap2*), neuron-specific nuclear protein (*NeuN*), neurofilament-L (*NF-L*), neurofilament-M (*NF-M*), neurofilament-H (*NF-H*), neuron-specific class III beta-tubulin (*Tuj-1*), nestin and growth-associated protein-43 (*GAP-43*), microtubule-associated protein tau (*MAPT*), and *Nestin*) mRNA (mRNA) compared with a reference gene, glyceraldehyde-3-phosphate dehydrogenase (*GAPDH*). Quantitative RT–PCR was performed using a custom-made RT–PCR assay with SYBR green chemistry (Toyobo) in an ABI Prism 7700 sequence detector (Applied Biosystems, Foster City, CA). The 2^-△△CT^ method was used to calculate the relative ratio of neuronal specific genes to *GAPDH* gene concentrations [16]. The sequences of the specific primers used are provided (Table 2).

**Table 2:** Primer sequences used for real-time quantitative RT–PCR

**Gene**	**Primer sequence**	**Product length (bp)**
Mtap2 for	catcatccgcactcctccaa	148
Mtap2 rev	aatcctaacctgacccccctt	
Nestin for	tcaaccctcaccactctatttt	143
Nestin rev	gctgttttctacttttacctctgtg	
NeuN for	caccactctcttgtccgtttg	106
NeuN rev	gctgctggctgagcatatct	
NF-L for	gaaggcgaagagaccagg	134
NF-L rev	gagcgagcagacatcaagtag	
NF-M for	accgaggcagaaggtgaag	137
NF-M rev	gatttgggcataggggattt	
NF-H for	ctcccaaaaattccctccata	139
NF-H rev	ctgtcactccttccgtcacc	
MAPT for	ccctggaggagggaataagaag	135
MAPT rev	aggtgccgtggagatgtgt	
Tuj-1 for	acgcatctcggagcagtt	125
Tuj-1 rev	cggacaccaggtcattca	
GAP-43 for	ggagaagaagggtgaagggg	100
GAP-43 rev	ggacggggagttatcagtgg	
GAPDH for	ccttccgtgttcctacccc	132
GAPDH rev	agcccaagatgcccttcag	
FRMD7 for	atgcaaggctttctggaagac	111
FRMD7 rev	cggaaactggaacctttgcta	

### Statistical analysis

All experiments were performed in triplicate for each condition, and from at least three different cell culture preparations. Results are expressed as mean±SEM. Statistical analysis was performed by Mann–Whitney U test or by Student *t* test using SPSS16.0 software (SPSS, Inc., Chicago, IL). Levels of significance were *p<0.05, **p<0.01, and ***p<0.001.

## Results

### Overexpression of FRMD7 promoted neurite outgrowth in RA-induced differentiated Neuro-2a cells

**Figure 1:**
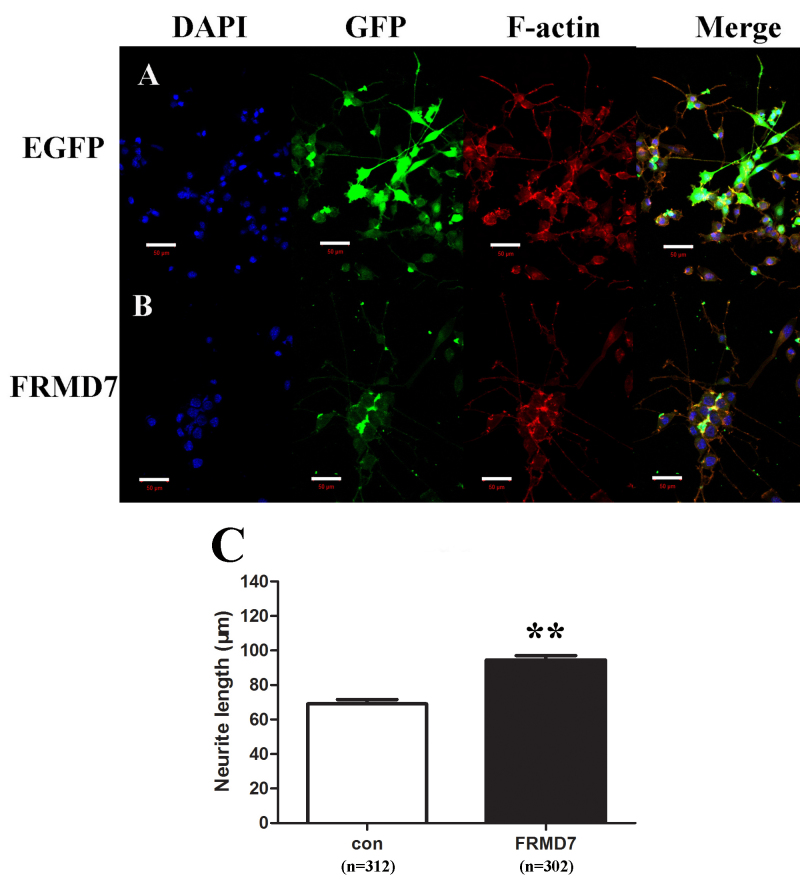
Effects of FRMD7 overexpression on neurite outgrowth in undifferentiated or differentiated Neuro-2a cells. **A**, **B**: Neuro-2a cells transfected with FRMD7-pEGFP-n1 (**B**) or the empty pEGFP-n1 vector (**A**) was treated with retinoic acid (RA) for 3 days. The cells were fixed and stained with TRITC-conjugated rhodamine-phalloidin. Statistics were gathered on the percentage of cells that bore neurites and the length of the longest outgrowing neurite from the cell bodies of the evaluated cells. FRMD7 demonstrated a remarkable potential to increase neurite length compared with control cells. However, the number of cells with neurites was no different between groups. The overexpression of FRMD7 without RA treatment did not change the morphology of Neuro-2a cells (data not shown). Magnification: 100x. Scale bars: 50 μm. **C**: The average length of the longest outgrowing neurite from differentiated Neuro-2a cells transfected with FRMD7-pEGFP-n1 (FRMD7) or the empty pEGFP-n1 vector (CON). **p<0.01 compared with the control.

Previous studies found that knockdown of *FRMD7* led to neurite outgrowth shortened during RA-induced differentiation in Neuro-2a cells [10]. However, whether FRMD7 can directly trigger the initiation of neurite extension and overexpression of FRMD7 promotes neurite outgrowth in Neuro-2a cells has not been elucidated. Thus, we sought to examine how overexpression of FRMD7 would affect neurite development in undifferentiated or differentiated Neuro-2a cells. We showed that overexpression of FRMD7 without RA treatment did not alter the morphology of Neuro-2a cells (data not shown). Conversely, cells transfected with FRMD7-EGFP-n1 showed a variety of morphological changes under RA-induced differentiated conditions. Data were gathered on the percentage of cells that bore neurites that were greater than one times the soma diameter from at least five randomly taken images per well. Neurite length was determined by measuring the length of the longest outgrowing neurite from the cell bodies of the evaluated cells. *FRMD7* transfection showed a remarkable ability to increase the length of neurites compared with the control cells under the same serum-depleted RA-induced conditions. We observed a significant increase in neurite length after *FRMD7* transfection (control=68.99±2.59, FRMD7=94.40±2.62, p<0.01). However, the number of cells with neurites was not significantly different (control 38.63±3.65, FRMD7 41.87±3.46; Figure 1 and Table 1).

### FRMD7 regulated mRNA expression levels of neuron-specific genes in Neuro-2a cells

**Figure 2:**
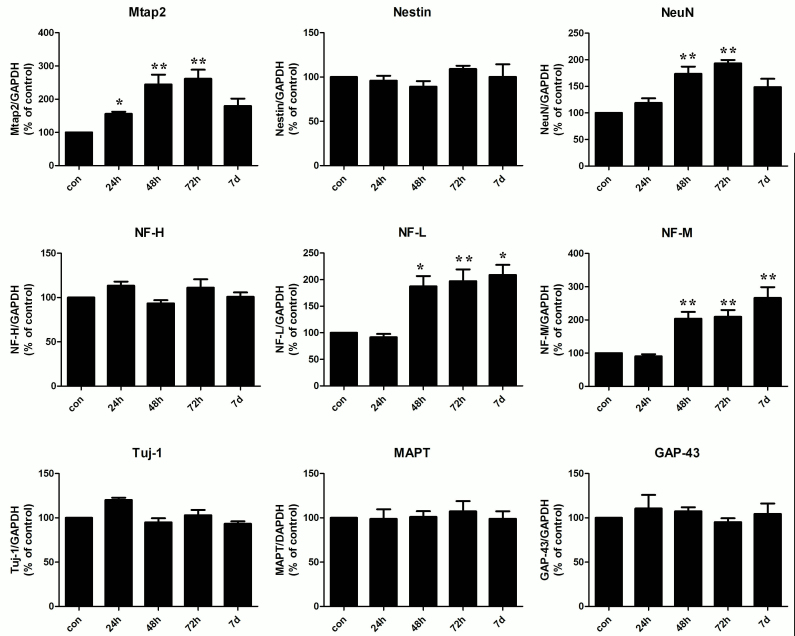
Overexpression of FRMD7 regulated the mRNA expression levels of neuron-specific genes in Neuro-2a cells. Cells transfected with the empty vector served as the negative control (MOCK group). The expression of *Mtap2*, *NF-L*, *NF-M*, and *NeuN* mRNA (mRNA) doubled 48 h after transfection. The expression levels of *NF-L* and *NF-M* mRNA continued to increase, reaching a 2.2-fold increase 7 days after transfection. The mRNA levels of other genes (*Nestin*, *NF-H*, *MAPT*, *GAP-43*, and *Tuj-1*) did not change obviously. The data are presented as changes relative to the MOCK group. All of the experiments were performed in triplicate, and the graph represents the average (columns, mean; bars, SEM; *p<0.05, **p<0.01 versus MOCK group).

**Figure 3:**
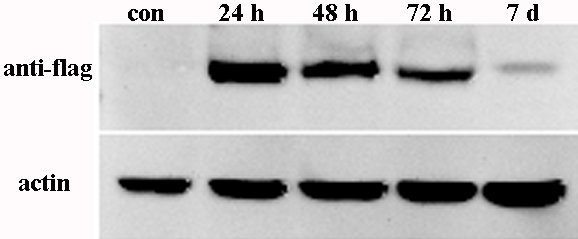
Expression levels of FRMD7 at the indicated time points in Neuro-2a cells were confirmed by western blot. FRMD7 was monitored by western blot in transfected Neuro-2a cells at the indicated time points. Samples (cells) from cultured Neuro-2a cells transfected with pcDNA3.1 (+)-FRMD7-flag at different time points (24 h, 48 h, 72 h, and 7 days) were detected. The blots were probed with the labeled antibody anti-flag. β-actin was used as an internal control.

To address the specific function of *FRMD7* in differentiation, we examined the mRNA expression levels of various neuron-specific genes related to different steps of neuronal development, including the cytoskeletal-related genes (*Mtap2*, *NF-L*, *NF-M*, *NF-H*, *Tuj-1*, and *MAPT*) and neuronal differentiation markers (*NeuN*, *GAP-43*, and *Nestin*). A time-course experiment was performed to determine the role of *FRMD7* in neuron-specific gene expression in Neuro-2a cells. Interestingly, within 48 h of transfection with FRMD7-pcDNA3.1, which contained a full-length cDNA encoding *FRMD7*, *Mtap2*, *NF-L*, *NF-M*, and *NeuN* mRNA expression levels significantly increased compared with the controls. The expression of *Mtap2*, *NF-L*, *NF-M*, and *NeuN* mRNA approximately doubled 48 h after transfection. The expression levels of *NF-L* and *NF-M* mRNA continued to increase, reaching a 2.2 fold increase 7 days after transfection (Figure 2). The expression levels of other genes that were tested did not change obviously. Furthermore, the expression levels of FRMD7 at the indicated time points in Neuro-2a cells were confirmed by western blot (Figure 3).

Mtap2 and neurofilaments (NFs) are neuronal cytoskeletal proteins that are expressed in mature neurons [17,18]. The above changes suggest that FRMD7 promotes neuronal differentiation and may be involved in the upregulation of cytoskeletal proteins, which affects not only F-actin but also microtubule and NF dynamics.

## Discussion

The *FRMD7* gene, which is the major etiological contributor of ICN, is a member of the super 4.1 family of proteins, and shares a 4.1 ezrin, radixin, moesin (FERM) domain at its N-terminus [4]. In a previous study, a significant reduction in the overall length of neurites was detected using RA-induced differentiation, when *FRMD7* was knocked down in Neuro-2a cells [10]. However, it remains unclear whether FRMD7 can trigger the initiation of neurite extension, and the precise biochemical role of FRMD7 in neuronal development has yet to be elucidated. To investigate the role of FRMD7 in the initiation of neurite outgrowth, we examined the differentiation of neuronal cells and neurite length when a plasmid containing a full-length cDNA coding for FRMD7 was transfected into Neuro-2a cells. We found that the overexpression of FRMD7 in Neuro-2a cells alone was not sufficient to induce the extension of neurites. However, following RA-induced neuronal differentiation of Neuro-2a cells, FRMD7 augmented neuronal formation, which resulted in longer neurites than in the control cells. This result was consistent with previous work [10] and further confirmed that FRMD7 plays a critical role in the process of RA-induced differentiation in Neuro-2a cells.

Neuro-2a is a mouse neural crest–derived cell line that has been extensively used to study neuronal differentiation. RA-induced neuronal differentiation in Neuro-2a cells is a complex procedure that alters the expression levels of many neuron-specific proteins. The sequence of neuronal differentiation events, including the loss of pluripotency, formation of neuronal precursors and their further maturation, were mirrored in the marker mRNA expression profiles. We performed a detailed series of time-lapse studies with 11 neuron-specific genes and their mRNA expression profiles after *FRMD7* transfection. The mRNA expression of *Mtap2*, NF genes (*NF-L* and *NF-M*) and NeuN were highly upregulated 48 h after transfection compared to control cells (p<0.05). Other genes showed no significant alteration. Expression of the housekeeping gene GAPDH was not statistically significantly different (p>0.05) between the different time points and treatments.

Mtap2 is a neuronal cytoskeletal protein expressed in mature neurons [17], which can interact with both microtubules [19] and actin filaments [20,21]. Mtap2 is an expressed abundantly during all stages of neuromorphogenesis [22]. In the primary hippocampal neurons, it is first expressed in neuroblasts before neurite initiation. It is then present in minor process that eventually differentiates into an axon. During later stages of differentiation, Mtap2 disappears from axons but is retained in dendrites [22]. This expression pattern suggests that Mtap2 plays a role in both early and late morphological events. As a result, FRMD7 may play an important role in neurite outgrowth involved in influencing the dynamics of F-actin [10,11]. Microtubules and F-actin are major structural components of the neuronal cytoskeleton, and play an essential role in the elaboration of axons and dendrites [20,21,23]. Our results suggest that FRMD7 regulates the neuronal differentiation in relation to Mtap2.

NFs are neuron-specific cytoskeletal intermediate filament proteins, which are assembled from three constituent polypeptides of apparent molecular weights of 68 kDa (NF-L), 145 kDa (NF-M), and 200 kDa (NF-H) as separated on a sodium dodecyl sulfate PAGE. They are known to undergo significant changes in subunit composition in both developing and adult neurons that inﬂuence both the morphology and physiology of axons [24,25]. NFs determine axonal caliber and promote axonal growth over long distances [26,27]. The NF-L and NF-M mRNAs are coexpressed during the early embryonic stages of neuronal differentiation and development, while *NF-H* mRNA is expressed only at a later embryonic and early postnatal stage [28]. The time course of NF mRNA expression in differentiating Neuro-2a cells was also examined. An increase in *NF-L* and *NF-M* mRNA levels was observed at early stage following the induction of differentiation. In contrast, the expression of *NF-H* mRNA followed a very different time course, with an increase later after differentiation [29]. FRMD7 upregulated the increase of *NF-L* and *NF-M* mRNA levels, but not *NF-H* in Neuro-2a cells, which suggests that FRMD7 promotes Neuro-2a cell differentiation at the early stage. It may also indicate that FRMD7 is involved in neuronal differentiation and development during the early stages of embryogenesis.

In summary, we have shown that FRMD7 promotes neurite outgrowth and may be involved in the movement of cytoskeletal proteins; this not only affects F-actin but also influences both NF and microtubule dynamics.
